# PM20D1 pode ser um Novo Garoto no Bairro na Estratificação de Risco Cardiovascular? Não Corra Antes de Poder Andar

**DOI:** 10.36660/abc.20220462

**Published:** 2022-08-25

**Authors:** Ana Teresa Timóteo

**Affiliations:** 1 Departamento de Cardiologia Hospital Santa Marta Centro Hospitalar Universitário Lisboa Central Lisboa Portugal Departamento de Cardiologia, Hospital Santa Marta, Centro Hospitalar Universitário Lisboa Central, Lisboa - Portugal; 2 NOVA Medical School Lisboa Portugal NOVA Medical School, Lisboa - Portugal

**Keywords:** PM20D1, Doenças Cardiovasculares, Doenças Neurodegenerativas, Obesidade, Diabetes Mellitus Tipo 2, Síndrome Metabólica, Doença de Alzheimer, Resistência a Insulina, Doença das Artérias Carótidas, Hipertensão

As doenças cardiovasculares são a principal causa de morte no mundo, representando cerca de 30% de todas as mortes, principalmente em países desenvolvidos, incluindo doenças cardiovasculares e cerebrovasculares.^1^ Além disso, na análise do relatório *Global Burden of Diseases 2019* , os fatores de risco cardiovascular representam 75% de toda a carga cardiovascular, principalmente a hipertensão, com importante impacto na mortalidade.^1^ Isso também é relevante nos países de língua portuguesa.^2^ A espessura média-intimal carotídea é reconhecida há muito tempo como um marcador substituto para doença arterial coronariana e tem um impacto prognóstico relevante.^3^ Por isso, é uma ferramenta útil na estratificação do risco cardiovascular.

N-acil aminoácidos (NAAA) são uma família de lipídios circulantes induzíveis pelo frio que estimulam a termogênese, e sua biossíntese em adipócitos marrons é mediada por uma enzima secretada chamada domínio Peptidase M20 contendo 1 (PM20D1).^4^ A regulação da atividade de PM20D1 e NAAA no plasma sanguíneo ainda é amplamente desconhecida.^4^ No entanto, o que já se sabe é que a PM20D1 circula em estreita associação com lipoproteínas de baixa e alta densidade que são poderosos coativadores da atividade da PM20D1 in vitro e a biossíntese de NAAA in vivo.^4^ A albumina sérica também é um carreador fisiológico de NAAA que separa NAAA de seus locais de produção, conferindo resistência à degradação hidrolítica e estabelecendo um equilíbrio entre frações termogênicas “livres” versus inativas “ligadas”.^4^

Anormalidades no gene PM20D1 têm sido associadas a diversas doenças, principalmente doenças neurodegenerativas, como Alzheimer e Parkinson.^5 , 6^ Há também associação com síndrome do ovário policístico.^7^ Em humanos, níveis séricos aumentados de PM20D1 e seus produtos catalíticos (NAAA) também estão associados à desregulação glicêmica relacionada à obesidade, resistência à insulina e síndrome metabólica e podem ser potencialmente usados como biomarcadores clínicos para diagnóstico e monitoramento desses distúrbios.^8^

O artigo publicado por Huang et al.^9^ no presente número de Arquivos Brasileiros de Cardiologia analisaram o papel da PM20D1 na aterosclerose carotídea (AC). Estudaram prospectivamente 231 pacientes com AC estabelecida (avaliada por ultrassonografia carotídea) e os compararam com o mesmo número de indivíduos saudáveis. Alguns pacientes do grupo AC foram avaliados no contexto de acidente vascular cerebral agudo. As características clínicas basais foram bem equilibradas entre os dois grupos. Como esperado, os pacientes com AC moderada a grave (incluindo aqueles com acidente vascular cerebral) apresentaram níveis mais elevados de marcadores inflamatórios, placas carotídeas mais instáveis e níveis mais elevados de colesterol LDL em comparação com indivíduos saudáveis e de gravidade leve a moderada. Os pacientes com AC apresentaram níveis mais baixos de PM20D1 em comparação com indivíduos saudáveis. Também, os pacientes com placas instáveis e AC mais grave apresentaram níveis significativamente mais baixos. Este biomarcador também está negativamente correlacionado com marcadores inflamatórios, mas não com perfis lipídicos. Nenhuma diferença foi encontrada de acordo com o índice de massa corporal. Este biomarcador apresentou boa acurácia discriminativa pela análise da curva ROC para AC (limite 5,4 ng/mL) e AC grave (3,99 ng/mL). Infelizmente, apenas relatam dados de regressão logística binária para as variáveis estudadas. Por esse motivo, não é possível confirmar com os dados disponíveis se este novo biomarcador é um preditor independente de desfecho e se tem valor prognóstico agregado em relação aos parâmetros clássicos. Além disso, os autores observaram que pacientes com níveis mais altos de PM20D1 apresentaram níveis mais baixos de colesterol LDL. No entanto, ao contrário do relato dos autores, não foi observada correlação significativa (coeficiente de correlação -0,071, p=0,126). Os autores não explicaram esse achado; se colocarmos em contexto, a relação entre eles está agora em questão. Por esse motivo, ainda existem importantes questões não respondidas sobre a interação entre os dois.

No geral, este estudo lançou alguma luz inicial sobre o possível papel dessa via na AC. No entanto, várias questões permanecem sem resposta, e também carece de informações adicionais sobre a aplicabilidade clínica do método, principalmente na prática diária, já que esse parâmetro ainda não está pronto para uso clínico.
