# Supplementary material for: PM20D1 pode ser um Novo Garoto no Bairro na Estratificação de Risco Cardiovascular? Não Corra Antes de Poder Andar
Source: Arq Bras Cardiol. 2022 Aug 25;119(3):380–1. [Article in Portuguese] doi: 10.36660/abc.20220462 (PMC9438542; doi:10.36660/abc.20220462)
Supplement: Translation

# Translation: Can PM20D1 be a New Kid on the Block in Cardiovascular Risk Stratification? Do Not Run before You Can Walk

**Authors:** Ana Teresa Timóteo

**Affiliations:** 1 Portugal Departamento de Cardiologia, Hospital Santa Marta, Centro Hospitalar Universitário Lisboa Central, Lisboa - Portugal; 2 Portugal NOVA Medical School, Lisboa - Portugal

**Keywords:** PM20D1, Cardiovascular Diseases, Neurodegenerative Diseases, Obesity, Diabetes Mellitus Type 2, Metabolic, Syndrome, Alzheimer Diseases, Insulin Resistance, Carotid Artery Diseases, Hypertension

Cardiovascular diseases are the leading cause of death worldwide, representing around 30% of all deaths, particularly in developed countries, including cardiovascular and cerebral-vascular diseases.^1^ Furthermore, in the analysis of the Global Burden of Diseases 2019 report, cardiovascular risk factors represent 75% of all cardiovascular burden, particularly hypertension, with an important impact on mortality.^1^ This is also relevant in Portuguese-speaking countries.^2^ Carotid intima-media thickness has long been recognized as a surrogate marker for coronary artery disease and has a relevant prognostic impact.^3^ For that reason, it is a useful tool in cardiovascular risk stratification.

N-acyl amino acids (NAAA) are a family of cold-inducible circulating lipids that stimulate thermogenesis, and their biosynthesis in brown adipocytes is mediated by a secreted enzyme called Peptidase M20 domain containing 1 (PM20D1).^4^ PM20D1 and NAAA activity regulation in blood plasma is still largely unknown.^4^ However, what is already known is that PM20D1 circulates in tight association with both low- and high-density lipoproteins that are powerful co-activators of PM20D1 activity in vitro and NAAA biosynthesis in vivo.^4^ Serum albumin is also a physiologic NAAA carrier that separates NAAA away from their sites of production, conferring resistance to hydrolytic degradation and establishing an equilibrium between thermogenic “free” versus inactive “bound” fractions.^4^ It has been hypothesized that lipoprotein particles are probably the main extracellular sites of NAAA biosynthesis, and this supports the concept that a lipoprotein-albumin network regulates the activity of circulating thermogenic lipid family.^4^

Abnormalities in the PM20D1 gene have been associated with several diseases, particularly neurodegenerative diseases, such as Alzheimer’s and Parkinson’s disease.^5 , 6^ There is also an association with polycystic ovarian syndrome.^7^ In humans, Increased serum levels of PM20D1 and its catalytic products (NAAA) are also associated with obesity-related glucose dysregulation, insulin resistance and metabolic syndrome and can be potentially used as clinical biomarkers for diagnosing and monitoring these disorders.^8^

The paper published by Huang et al.^9^ in the present issue of *Arquivos Brasileiros de Cardiologia* studied the role of PM20D1 in carotid atherosclerosis (CA). They prospectively studied 231 patients with established CA (assessed by carotid ultrasound) and compared them with the same number of healthy individuals. Some patients in the CA group were assessed in the context of acute stroke. Baseline clinical characteristics were well balanced between both groups. As expected, patients with moderate to severe CA (including those with stroke) had higher levels of inflammatory markers, more unstable carotid plaques and higher LDL cholesterol levels compared with mild to moderate severity and healthy individuals. Patients with CA had lower levels of PM20D1 compared to healthy individuals. Also, patients with unstable plaques and more severe CA had significantly lower levels. This biomarker is also negatively correlated with inflammatory markers but not lipid profiles. No difference was found according to body mass index. This biomarker showed good discriminative accuracy by ROC curve analysis for CA (cut-off 5.4 ng/mL) and severe CA (3.99 ng/mL). Unfortunately, they only report binary logistic regression data for the studied variables. For that reason, it is not possible to confirm with the available data whether this new biomarker is an independent predictor of outcome and if it has added prognostic value compared to the classical parameters. Also, the authors observed that patients with higher levels of PM20D1 had lower LDL cholesterol levels. However, contrary to the authors’ report, no significant correlation was observed (correlation coefficient -0.071, p=0.126). The authors did not explain this finding; their relationship is now in question if we put it into context. For that reason, there are still important unanswered questions regarding the interaction between both players.

Overall, this study shed some initial light on the possible role of this pathway for CA. However, multiple questions remain unanswered, and it also lacks additional information on the clinical applicability of the method, particularly in daily practice, because this parameter is not yet ready for its clinical use.
